# Radiologists can visually predict mortality risk based on the gestalt of chest radiographs comparable to a deep learning network

**DOI:** 10.1038/s41598-021-99107-0

**Published:** 2021-10-01

**Authors:** Jakob Weiss, Jana Taron, Zexi Jin, Thomas Mayrhofer, Hugo J. W. L. Aerts, Michael T. Lu, Udo Hoffmann

**Affiliations:** 1 Artificial Intelligence in Medicine (AIM) Program, Brigham and Women’s Hospital, Harvard Medical School, Harvard Institutes of Medicine (HIM), Suite 343, 77 Avenue Louis Pasteur, Boston, MA 02115 USA; 2 Cardiovascular Imaging Research Center, Massachusetts General Hospital, Harvard Medical School, Charles River Plaza, 165 Cambridge Street, Boston, MA 02114 USA; 3 Department of Radiation Oncology and Dana-Farber Cancer Institute, Harvard Medical School, 75 Francis Street and 450 Brookline Avenue, Boston, MA 02115 USA; 4 Department of Radiology, Brigham and Women’s Hospital and Dana-Farber Cancer Institute, Harvard Medical School, 75 Francis Street and 450 Brookline Avenue, Boston, MA 02115 USA; 5 Department of Diagnostic and Interventional Radiology, University Hospital in Freiburg im Breisgau, Hugstetter Str. 55, 79106 Freiburg, Germany; 6 School of Business Studies, Stralsund University of Applied Sciences, Zur Schwedenschanze 15, 18435 Stralsund, Germany; 7 Radiology and Nuclear Medicine, CARIM and GROW, Maastricht University, Universiteitssingel 40, 6229 ER Maastricht, The Netherlands

**Keywords:** Radiography, Prognostic markers

## Abstract

Deep learning convolutional neural network (CNN) can predict mortality from chest radiographs, yet, it is unknown whether radiologists can perform the same task. Here, we investigate whether radiologists can visually assess image gestalt (defined as deviation from an unremarkable chest radiograph associated with the likelihood of 6-year mortality) of a chest radiograph to predict 6-year mortality. The assessment was validated in an independent testing dataset and compared to the performance of a CNN developed for mortality prediction. Results are reported for the testing dataset only (n = 100; age 62.5 ± 5.2; male 55%, event rate 50%). The probability of 6-year mortality based on image gestalt had high accuracy (AUC: 0.68 (95% CI 0.58–0.78), similar to that of the CNN (AUC: 0.67 (95% CI 0.57–0.77); p = 0.90). Patients with high/very high image gestalt ratings were significantly more likely to die when compared to those rated as very low (p ≤ 0.04). Assignment to risk categories was not explained by patient characteristics or traditional risk factors and imaging findings (p ≥ 0.2). In conclusion, assessing image gestalt on chest radiographs by radiologists renders high prognostic accuracy for the probability of mortality, similar to that of a specifically trained CNN. Further studies are warranted to confirm this concept and to determine potential clinical benefits.

## Introduction

Chest radiography is the most commonly performed medical imaging study in the United States^1^. Often, the primary purpose of standard clinical interpretation of chest radiographs is the diagnosis of findings such as pneumonia that may explain the leading clinical symptoms (i.e., fever or shortness of breath). However, with the trend towards more structured radiology reports, other abnormalities such as enlarged heart size or degenerative bone changes^2^ unrelated to primary symptoms are reported as well. Most of these findings have prognostic rather than diagnostic value. For example, cardiac silhouette enlargement is associated with an increased risk of coronary events^3^ and low bone density may serve as a predictor for hip fractures^4^.

With the advance of artificial intelligence—specifically deep learning (DL) techniques—into medicine, there has been an increasing interest to use these techniques to derive prognostic information from medical images that have traditionally been considered for diagnostic purposes^5,6^. For example, DL Convolutional Neural Networks (CNNs) have been successfully deployed to analyze medical images and predict treatment response of lung cancer (from CT images)^7^, biological brain age as a biomarker for dementia (from MR images)^8^ or long-term mortality (from chest radiographs)^9^. A limitation of this type of analysis is the clinical interpretation of the results and the justification of potential treatment decisions^10^ since the CNN decision-making process remains largely unknown^9^.Yet, this is similar to physicians intuitively estimating health or frailty of a patient based on their overall appearance or gestalt instead of relying on specific test results. Translating this concept to radiological image interpretation would mean to determine a patients overall condition based on the entire image information or image gestalt^11^ defined as the degree of deviation from a completely unremarkable image. This concept is supported by studies showing that a DL-based general assessment of an entire chest radiograph outperforms individual findings reported by radiologists for predicting mortality^9^.Thus, our goal was to investigate whether radiologists can perform a similar task and explore how this is accomplished. Here, we present a pilot study to determine whether radiologists can be trained to predict mortality from chest radiographs and compare the predictive performance to a DL CNN developed for the same task^9^. This may path the way for future studies to investigate the potential clinical use of this concept.

## Methods

### Study design and population

For this retrospective secondary use study, chest radiographs were drawn from the screening radiography arm of participants enrolled in the National Lung Screening Trial (NLST) on American College of Radiology Imaging Network (ACRIN) sites^12^. The NLST enrolled a community-based cohort (age 55–74) of current or former heavy smokers (smoking cessation ≤ 15 years) with a ≥ 30 pack-year smoking history who were randomized to either low-dose chest CT or chest radiograph for lung cancer screening between 2002 and 2004.

All participants provided written informed consent for NLST. In addition, secondary use of data was approved by the Partners Human Research Committee at the Massachusetts General Hospital as well as the American College of Radiology Imaging Network (ACRIN). Moreover, all methods and analyses were carried out in accordance with relevant guidelines and regulations.

### Image analysis

**Figure 1:**
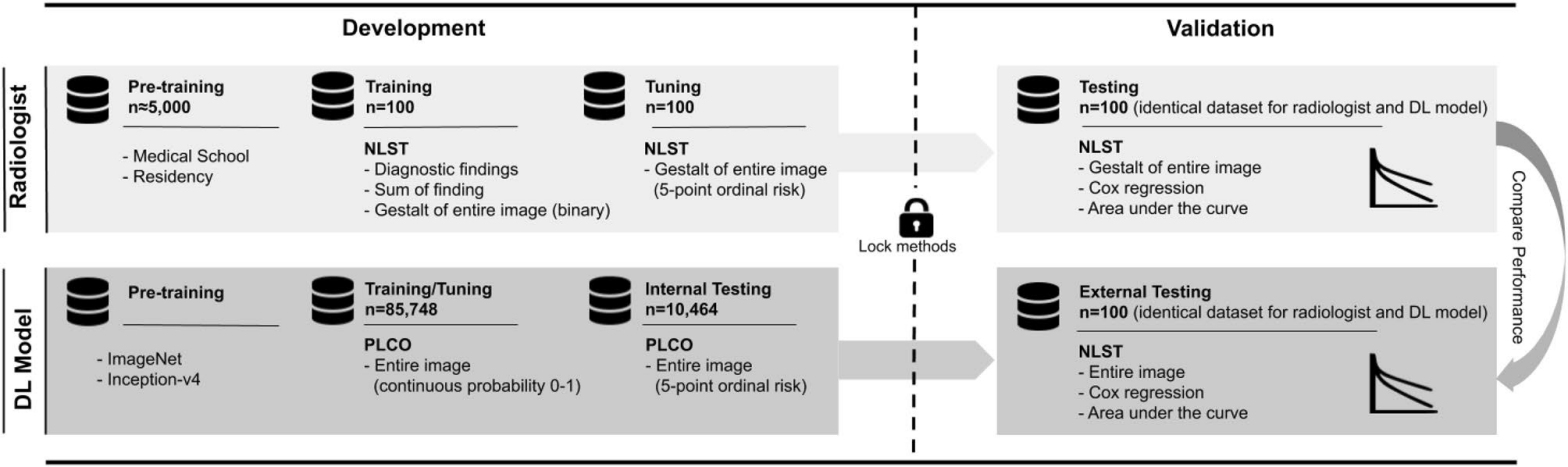
Overview of the study design. *DL* deep learning, *NLST* National Lung Screening Trial, *PLCO* Prostate, Lung, Colorectal, and Ovarian Cancer Screening Trial.

For image analysis, we adopted a framework known from machine learning research and divided the data into training, tuning, and testing sets (Fig. 1). Each set consisted of 100 participants with a 50% mortality rate. All assessments were made in consensus by two radiologists (JW and JT; final year resident and board-certified radiologist) with 6 and 7 years of experience and more than 5000 diagnostic interpretations of chest radiographs. Study reads were performed using the original DICOM images (OsiriX version 11, Pixmeo SAR, Bernex, Switzerland) in independent training, tuning and testing sessions (as described below) at least two weeks apart. The primary outcome was 6-year mortality risk. Reference standard for all ratings was vital status as reported in NLST and verified via death certificates and the National Death Index^12^ after 6-year follow-up.

#### Training data set

In a first step, the images were assessed for the presence of a predefined set of findings: (a) standard diagnostic findings typically reported in clinical assessment (i.e., suspect pulmonary lesion, emphysema, cardiac enlargement, aortic pathology), and (b) any abnormality present in the scan including ‘subclinical’ findings (i.e., pleural fibrosis, aortic calcification, or degenerative changes of the skeleton; for a summary of all diagnostic and subclinical findings refer to Supplementary Table S1).

In a second step, radiologists assessed the gestalt of the entire image, defined as the degree of deviation from an unremarkable chest radiograph associated with the likelihood of 6-year mortality.

#### Tuning data set

Based on the experiences of training, the reading was performed using the image gestalt to predict a participant’s likelihood of mortality. Because binary grading carries the biggest loss of information, for tuning, the rating scheme for the ‘image gestalt’ was changed from binary to a 5-point ordinal scale (1 = very low, 2 = low, 3 = moderate, 4 = high, 5 = very high deviation from normal and associated with the likelihood of 6-year mortality). The initial interpretation of the chest radiographs was blinded followed by an unblinding and a review of the images with the knowledge the participant’s vital status.

#### Testing data set

For testing, a third independent and held out data set was selected. Based on the results of training and tuning, the likelihood ratings of mortality were based on the image gestalt and the 5-point ordinal scale. Radiologists were blinded to any information about the participants, including outcome or cardiovascular risk factors.

### Statistical analyses

Demographics and study related outcomes are given in mean ± standard deviation or absolute/relative frequencies and were compared using Fisher’s exact test for categorical variables and ANOVA for continuous variables. The primary outcome was 6-year mortality for all ratings.

In the training data set, radiologists assessed traditional diagnostic findings, subclinical findings, and the image gestalt on a binary scale (present/absent). For further analysis, the presence of a clinically relevant finding (defined as presence of ≥ 1 traditional diagnostic finding), the sum of diagnostic plus subclinical findings, and image gestalt were used as predictors.

In the tuning and testing data set, image gestalt was treated as an explanatory variable assessed on a 5-point ordinal scale.

To determine the association of explanatory variables and primary outcome, we estimated hazard ratios (HR) with 95% confidence intervals (CI) by constructing Cox proportional hazard models. The discriminatory ability was assessed by calculating area under the receiver operating characteristic curves (AUCs).

To set these results in context, we compared the HRs and AUCs of radiologists to those of a recently published DL CNN developed for mortality prediction^9^ using DeLong test^13^. DL predictions were based on a probability score from 0 to 1, which was stratified into five risk groups (very low, low, moderate, high, very high risk) based on quantiles using the following thresholds (0; 0.063583; 0.096197; 0.1579735; 0.34843)^9^. For correlation, both ratings (radiologists and DL CNN model) were grouped into ‘high risk’ (moderate, high and very high) and ‘low risk’ (very low and low) and compared by absolute/relative frequencies and descriptive baseline characteristics using Fisher´s exact test for categorical variables and student´s t-test for continuous variables.

All statistical analyses were performed using STATA (Version 15.1, StataCorp, College Station, TX). All p values were 2-sided and considered to indicate statistical significance < 0.05.

## Results

### Study population

Patient demographics and clinical information for the training, tuning and testing data sets are shown in Table 1. Overall, there were no clinically meaningful differences in demographics, cardiovascular risk factors, history of stroke, myocardial infarction, cancer or length of follow-up between the data sets.

**Table 1:** Patient characteristics in the training, tuning and testing dataset.

Variables	Training	Tuning	Testing	*p*
	*N (%)/mean* ± *SD (IQR)*	*N (%)/mean* ± *SD (IQR)*	*N (%)/mean* ± *SD (IQR)*	
Participants	100	100	100	1.00
**Race**				
White	96 (96%)	97 (97%)	86 (86%)	0.008
Black	3 (3%)	3 83%)	12 (12%)	0.01
Other	1 (1%)	–	2 (2%)	0.8
Male sex	58 (58%)	61 (61%)	55 (55%)	0.7
Age	62.6 ± 5.6	63.6 ± 5.7	62.5 ± 5.2	0.3
Obesity (BMI ≥ 30 kg/m^2^)	20 (20%)	30 (30%)	25 (25%)	0.3
**Smoking**				
Current	52 (52%)	49 (49%)	55 (55%)	0.3
Former	48 (48%)	51 (51%)	45 (45%)	0.7
Diabetes	9 (9%)	14 (14%)	14 (14%)	0.5
Hypertension	42 (42%)	42 (42%)	34 (34%)	0.4
**History**				
Stroke	9 (9%)	5 (5%)	6 (6%)	0.5
Myocardial infarction	7 (7%)	14 (14%)	18 (18%)	0.06
Cancer	4 (4%)	4 (4%)	5 (5%)	1.00
Median follow-up (years)	5.9 (4.1–6.4)	5.9 (3.5–6.5)	5.9 (3.4–6.4)	0.9

Baseline characteristics and risk factors of the participants included for training, tuning and testing.

*IQR* interquartile range, *BMI* body mass index calculated as weight in kg/height in meters squared.

### Training data set

#### Assessment of diagnostic findings

**Figure 2:**
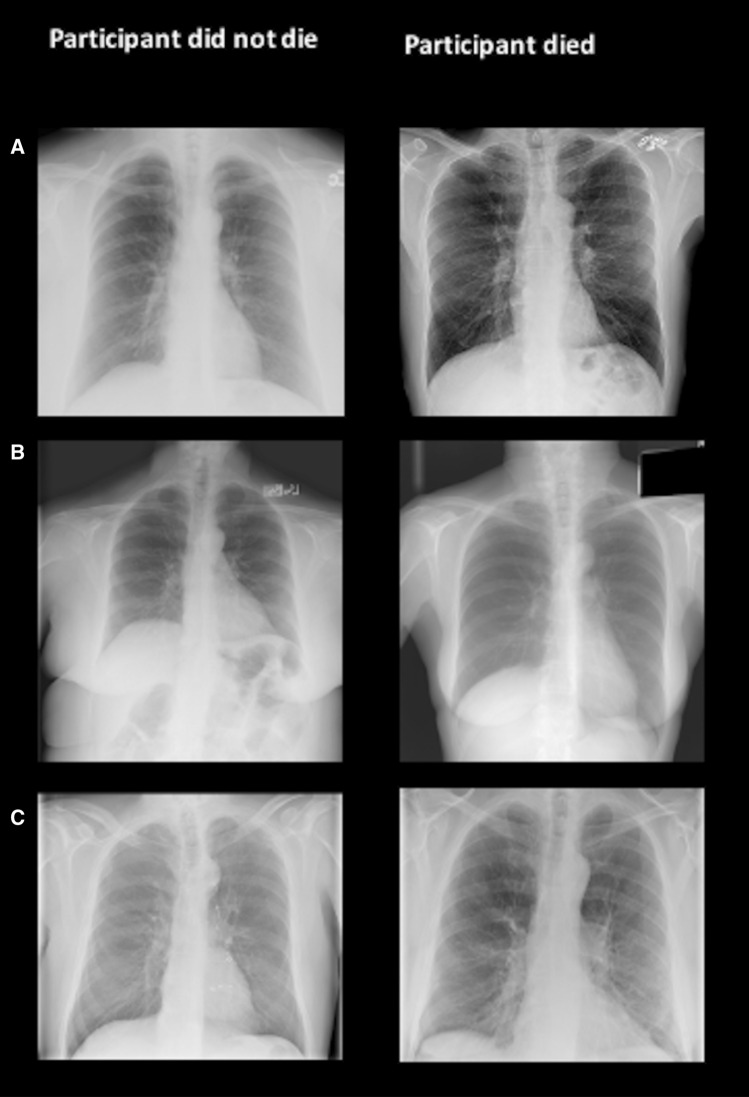
Representative cases from the training data set: in the training data set, image findings (diagnostic and subclinical) as well as gestalt of the image (defined as the degree of deviation from an unremarkable chest radiograph associated with the likelihood of 6-year mortality rated on a binary scale) were assessed. In row (**A**), both participants presented with emphysema, indicating that a single major diagnostic finding are not reliable predictors for outcome. In row (**B**), 13 findings were reported in image on left, 4 findings were reported in image on right; example that sum of findings per subject was not associated with mortality. In row (**C**), image on left surgical clips indicate elevated probability of dying, however, radiologists rated gestalt of the image as “absent” on left and as “present” on right.

The most frequent individual diagnostic findings were emphysema (18%), suspicious lung nodules (9%), aortic pathologies (9%) and cardiac enlargement (8%). There was no significant association between the presence of individual diagnostic findings and mortality (HR: 0.92 (95% CI 0.60–1.44); p = 0.74) (Fig. 2a). Similarly, the number of all reported findings per subject (median: n = 9; IQR 7; 11) was not associated with mortality (HR 1.03 (95% CI 0.95–1.11); p = 0.52) (Fig. 2b).

#### Image gestalt

Treating the image gestalt as a binary outcome, a rating of ‘high’ was associated with a HR of 1.83 (95% CI 1.05–3.19) which was significantly higher as compared to a rating of ‘low’ (p = 0.03).

The discriminatory ability for predicting 6-year mortality was modest (AUC: 0.60 (95% CI 0.51–0.69) (Fig. 2c).

As a result, the image gestalt was chosen as the primary method to predict mortality from chest radiographs.

### Tuning data set

In the tuning process, to increase information gain from ‘image gestalt’-ratings, we revised the rating scale from binary to ordinal (1 = very low, 2 = low, 3 = moderate, 4 = high, 5 = very high). This change resulted in a stronger association and improved discrimination. For example, participants with a chest radiograph with the highest ratings were six times more likely to die than those assessed as very low (Supplementary Figure S1). Similarly, the discriminatory ability increased as compared to the binary rating scale (AUC: 0.60 (95% CI 0.51–0.69) vs. 0.69 (95% CI 0.59–0.79); respectively; p = 0.19)). Thus, the 5-point rating scale for image gestalt was the method of choice to predict mortality from chest radiographs for testing.

### Testing data set

**Figure 3:**
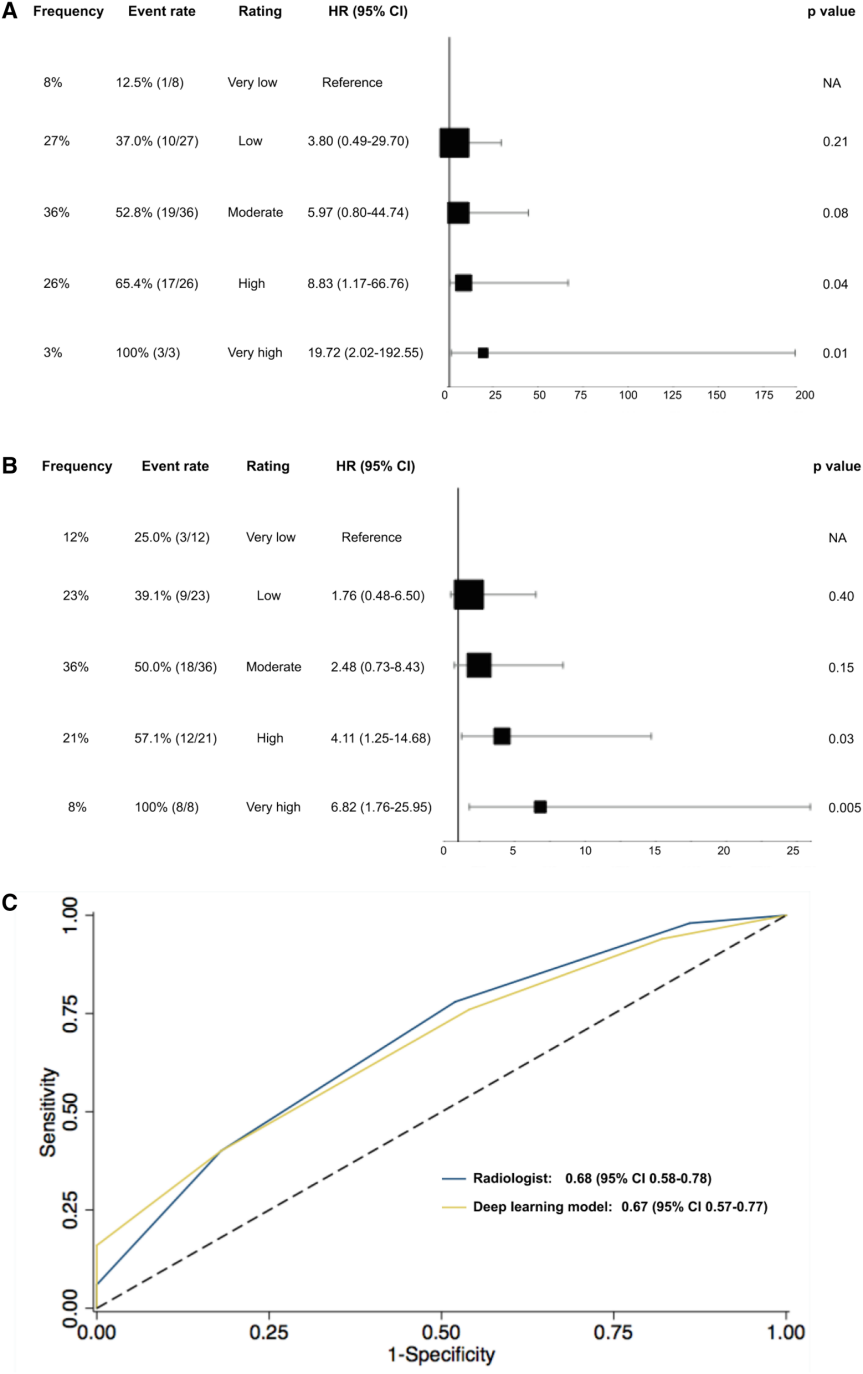
Gestalt ratings for 6-year mortality by radiologists (**A**) and the deep learning network (**B**) in the testing dataset as well as the areas under the curve for the discriminatory ability (**C**). *HR* hazard ratio, *CI* confidence interval.

In the testing data set, radiologists rated 8% of participants as having a very low gestalt rating and thus likelihood of dying, 27% as low, 36% as moderate, 26% as high and 3% as very high. The proportion of events increased within these ratings from 12.5% (n = 1) in the very low to 100% (n = 3) in the very high group. The HR for high (HR 8.83 (1.17–66.76); p = 0.04) and very high gestalt ratings (HR 19.72 (2.02–192.55); p = 0.01; Fig. 3a) was significantly higher as compared to patients with very low ratings. The AUC was 0.68 (95% CI 0.58–0.78) and similar to the AUC derived from the tuning data set (AUC: 0.69 (95% CI 0.59–0.79); p = 0.87).

### Comparison of radiologists and DL CNN in the testing data set

**Figure 4:**
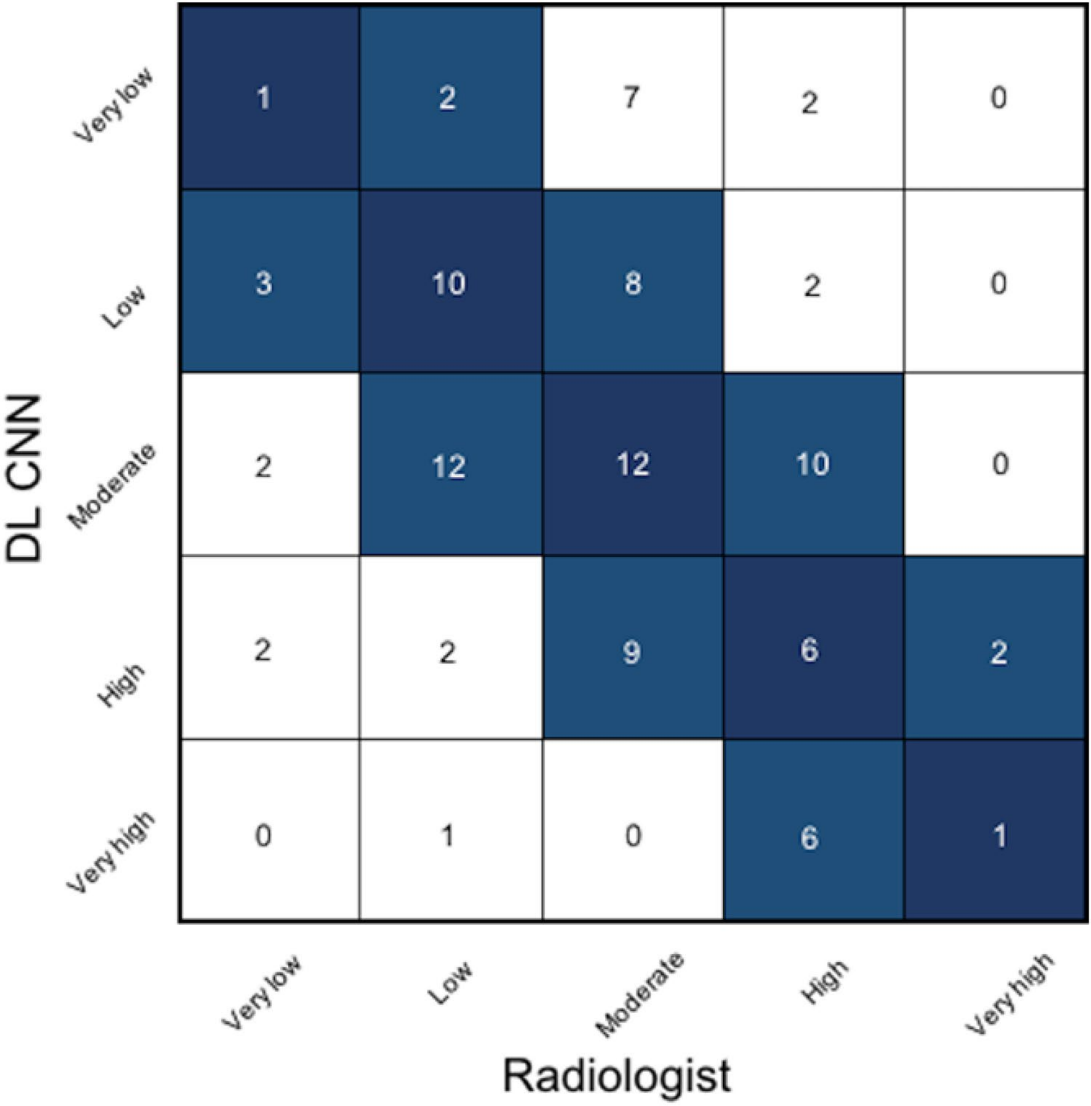
Confusion matrix of the risk ratings for 6-mortality between radiologists and the deep learning convolutional neural network. Dark blue: agreement between radiologists and the deep learning convolutional neural network; light blue: agreement with deviation by one category. *DL CNN* deep learning convolutional neural network.

Overall, the gestalt ratings for the likelihood of dying within 6 years by radiologists and the DL CNN showed agreement (defined as being in the same risk group or deviation by one category) in 82% of patients (Fig. 4). Moreover, the proportion of patients in each group was not significantly different between the radiologists and the DL CNN (8% and 12% very low, 27% and 23% low, 36% and 36% moderate, 26% and 21% high and 3% and 8% very high; respectively; p ≥ 0.21). Likewise, event rates across groups did not differ significantly between radiologists and the DL CNN (12.5% vs. 25% for very low; 37% vs. 39.1% for low; 52.8% vs. 50% for moderate ratings; 65.4% vs. 57.1% for high; 100% each for very high ratings; p ≥ 0.62; Fig. 3b). Similar to the radiologists’ ratings, HR increased by risk groups reaching statistical significance for high (HR 4.11 (1.25–14.68); p = 0.03) and very high gestalt ratings (HR 6.82 (1.79–25.95); p = 0.005; Fig. 3b). Consistent with these findings, the discriminatory ability of radiologists and the DL CNN was similar (AUC: 0.68 (95% CI 0.58–0.78) vs. 0.67 (95% CI 0.57–0.77); p = 0.90, Fig. 3c).

**Figure 5:**
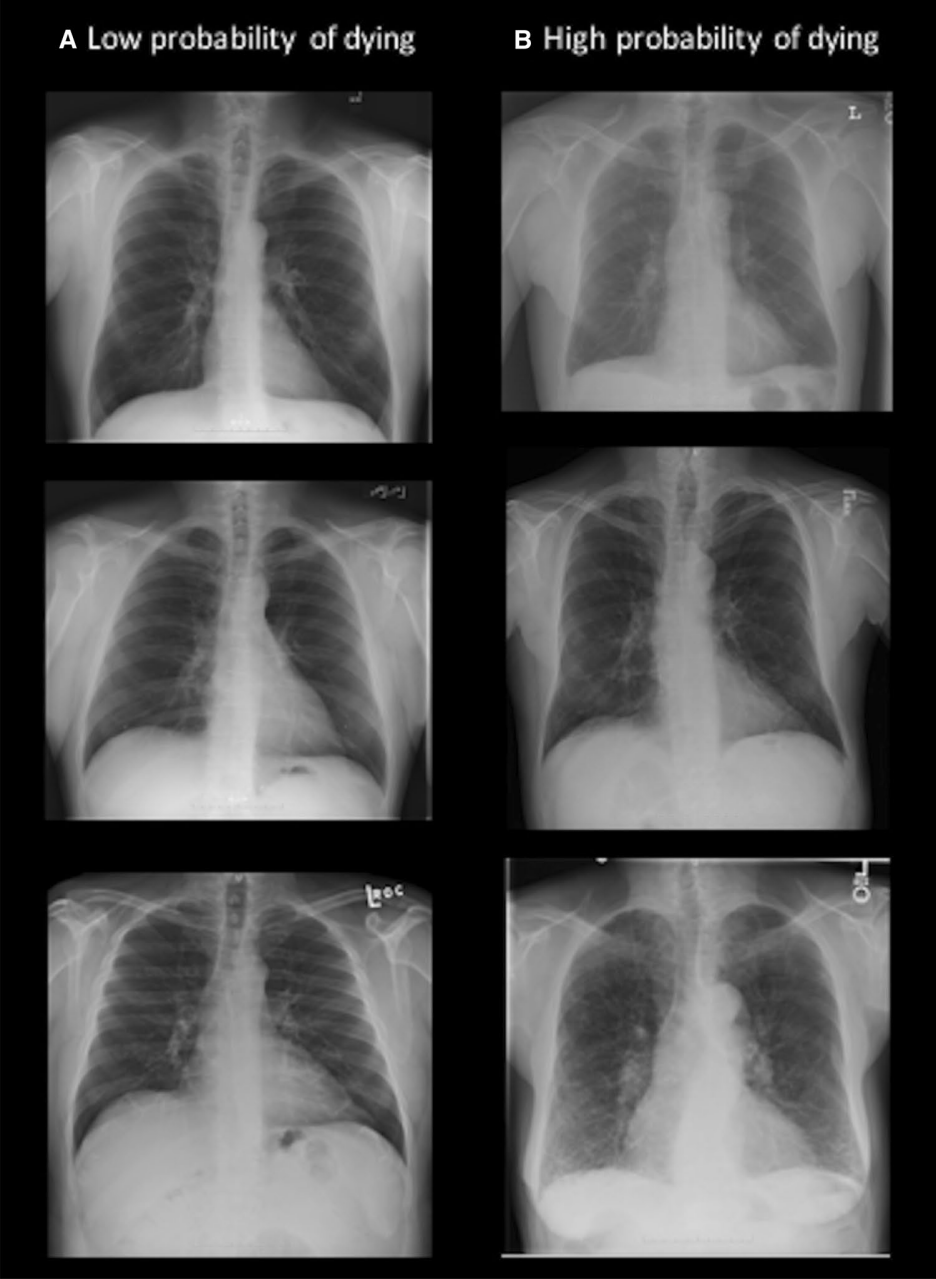
Representative images of participants correctly classified as low (**A**) and high (**B**) risk of dying by radiologists and the deep learning convolutional neural network.

In a next step, gestalt ratings for the likelihood of dying were dichotomized into a high (very high, high and moderate ratings) and low (low and very low ratings) risk group to enable a meaningful comparison of patient characteristics (Supplementary Table S2). Radiologists identified 65% (65/100) participants at high risk and 35% (35/100) at low risk for 6-year mortality. Comparing participants classified into high and low risk by radiologists, baseline characteristics were not significantly different (p ≥ 0.34), but a tendency towards a higher risk profile comprised of higher age, obesity and a higher prevalence of diabetes and hypertension in those at high risk was observed. All image findings appeared in the high-risk group, reaching statistical significance for the prevalence of cardiac enlargement (high vs. low risk group 13.9% vs. 0%; p = 0.03). Similarly to the radiologists, the DL CNN identified 65% (65/100) of participants at high risk and 35% (35/100) at low risk for 6-year mortality with a comparable high-risk profile in high risk participants. In direct comparison of both risk groups classified by radiologists vs. DL CNN, no differences were found in baseline characteristics and imaging findings (all p values ≥ 0.12) (Supplementary Table S2, representative images Fig. 5).

Radiologists vs. DL CNN correctly classified 78% (39/50) vs. 76% (38/50) as high risk participants (with 60% (30/50) concordance) and 48% (24/50) vs. 46% (23/50) as low risk participants (with 26% (13/50) concordance). In those correctly classified as high risk of dying, baseline characteristics and image findings had a similar tendency towards a high-risk profile (Supplementary Table S3).

## Discussion

In this pilot study, we demonstrate that radiologists can be trained to predict mortality from chest radiographs based on image gestalt with similar predictive performance to a DL CNN developed for the same task^9^. This is reflected in strong agreement of over 80% between radiologist and DL CNN-based predictions of mortality, assigning participants concordantly into one of five ordinal risk categories represented by increasing survival rates. Radiologists’ rating of gestalt as a proxy for the probability of survival was not associated or explained by traditional diagnostic imaging findings, patient demographics or risk factors, suggesting that gestalt as a whole is more than the sum of its individual parts^14^ and may constitute an additional and independent prognostic tool.

Translated into medical practice, assessing the gestalt is the ability to combine a multitude of patient information into one general impression (e.g., physical and mental condition, gait, ability to communicate, etc.). For some, this is considered the very essence of being an experienced physician. Our pilot study confirms that the same principles can be applied to radiological images and that using the gestalt of a chest radiograph—instead of the individual prognostic and diagnostic findings—renders an independent prognostic assessment. Although the literature on this is scarce, the existence of an underlying signal of abnormality independent of spatial information has been reported before^15^. For example, Brennan et al. demonstrated that radiologists had the ability to identify patients mammograms as likely to develop breast cancer before any objective signs of cancer became apparent^11^.

It is also remarkable that the predictive performance of the image gestalt assessments was comparable to that of a DL CNN specifically trained to predict mortality on more than 80.000 images. Although not perfect, our predictive accuracy (AUC: 0.68) is well within the range of clinically accepted risk prediction scores, including the Framingham risk score or ASCVD risk score for cardiovascular events and lung cancer risk prediction models which achieve AUCs around 0.7^16^. Not surprisingly, more specific morphological markers, for example the presence and extent of coronary artery calcium, a marker for subclinical atherosclerosis, may achieve higher predictive ability with AUC values around 0.8^17^.

One of the main drawbacks of DL CNN-based prognostic assessments is the inability to reconstruct the CNN decision-making process^9^ and the subsequent lack to support or justify specific changes in patient management^10^. In respect to decoding the interpretation, it turns out that the interpretation of image gestalt performed by radiologists is not much different. In our study, the assignment to one of the risk categories could not be explained by differences in patient demographics (sex, age, race) or clinical risk factors, although those classified as high risk were more likely men, tended to be older and showed a higher prevalence of cardiovascular risk factors and image findings. While this is not surprising, future research is warranted to define what contributes to and guides image gestalt ratings and how the human brain differentiates the interpretation of specific findings from the interpretation of gestalt. Until then, similar to DL CNN findings, the recommendations based on these assessments will remain unspecific.

There are limitations to our study. The main purpose of our study was to compare visual assessment of chest radiographs by radiologists with a deep learning model for predicting mortality. However, as the training, tuning and testing dataset were highly enriched for events (event rate 50%), our results cannot be used to make any conclusions about the strength of the performance (i.e. high) and the potential clinical use. To answer this question, future studies evaluating the potential clinical benefit need to include cohorts that reflect clinical event rates. In addition, although predicting mortality is not a clinical task and no radiologist is formally trained in this it remains unknown whether experience in clinical training and the approach how to read a chest radiograph (i.e. which regions are studied) is relevant for individual performance. Therefore, future studies should focus on a larger group of independent readers with different levels on experience to investigate this in detail. Moreover, the comparison to the DL-CNN is limited because the gestalt rating of the radiologists was subjectively defined using an ordinal scale, whereas the DL CNN used fixed cut-off values of the continuous model output. Nevertheless, the fact that humans trained on 200 chest radiographs achieved a similar accuracy as a DL-CNN trained on more than 80,000 is in itself remarkable and facilitated the best approach for pairwise comparison. Finally, this study lacks the comparison to other deep learning architectures.

In conclusion, radiologists can visually predict mortality risk based on the gestalt but not diagnostic findings of chest radiographs with similar accuracy as a specifically trained deep learning network. Further studies are warranted to confirm this concept in larger representative patient populations and to determine potential clinical benefits. In addition, studying the underlying neurological basis for gestalt interpretation may provide more insights about the nature of this assessments and may improve clinical interpretability.

## Supplementary Information

Supplementary Information.

## Data Availability

Imaging and demographic from NLST can be obtained through the NHI/NCI Cancer Access Data System.
