# Supplementary material for: Radiologists can visually predict mortality risk based on the gestalt of chest radiographs comparable to a deep learning network
Source: Sci Rep. 2021 Oct 1;11:19586. doi: 10.1038/s41598-021-99107-0 (PMC8486799; doi:10.1038/s41598-021-99107-0)
Supplement: Supplementary file 1 — Supplementary Information. [file 41598_2021_99107_MOESM1_ESM.pdf]

Supplemental Data to: Image gestalt of chest radiographs predicts long-term mortality in patients at risk for lung cancer: a comparative study between radiologists and a deep learning network

Jakob Weiss, MD[1,2,3,4,5*], Jana Taron, MD[2,5*], Zexi Jin[2], Thomas Mayrhofer, PhD[2,6], Hugo J.W.L. Aerts, PhD[1,2,3,4,7], Michael T. Lu, MD, MPH[1,2], Udo Hoffmann, MD, MPH[1,2]

[1]Artificial Intelligence in Medicine (AIM) Program, Brigham and Women's Hospital, Harvard Medical School, Harvard Institutes of Medicine (HIM), 77 Avenue Louis Pasteur, Boston, MA 02115, United States of America.

[2]Cardiovascular Imaging Research Center, Massachusetts General Hospital, Harvard Medical School, Charles River Plaza, 165 Cambridge Street, Boston, MA 02114, United States of America.

Departments of [3]Radiation Oncology and [4]Radiology, Brigham and Women's Hospital and Dana-Farber Cancer Institute, Harvard Medical School, 75 Francis Street and 450 Brookline Avenue, Boston, MA 02115, United States of America.

[5]Department of Diagnostic and Interventional Radiology, University Hospital in Freiburg im Breisgau, Hugstetter Str. 55, 79106 Freiburg, Germany

[6]School of Business Studies, Stralsund University of Applied Sciences, Zur Schwedenschanze 15, 18435 Stralsund, Germany.

[7]Radiology and Nuclear Medicine, CARIM & GROW, Maastricht University, Universiteitssingel 40, 6229 ER Maastricht, The Netherlands.

Supplemental Tables

Supplemental Table S1
Supplemental Table S2
Supplemental Table S3

Supplemental Figures

Supplemental Figure S1

Supplemental Tables

**1a. Training step 1**		**Findings**
Diagnostic findings likely to cause death within next 6 years		• *Suspect pulmonary nodule*<br>• *Emphysema*<br>• *Cardiac enlargement*<br>• *Aortic pathology*

**1b. Training step 2**	**Site/Organ**	**Findings**
Subclinical findings without immediate consequences for clinical management	*Soft tissue*<br>• *Chest*<br>• *Abdomen*	• Breast shadow abnormality<br>• Soft tissue lesions<br>• Adipose tissue<br>• Implants
	Trachea	• *Tracheal shift*<br>• *Tracheal bending*<br>• *Calcification*
	Pleura<br>• *Left*<br>• *Right*	• *Effusion*<br>• *Fibrosis/Thickening*<br>• *Calcifications*<br>• *Pleural lesion*
	Lungs<br>• *Left*<br>• *Right*	• *Pulmonary mass (>3cm)*<br>• *Pulmonary nodule (<3cm)*<br>• *Granuloma/benign nodule/benign calcification*<br>• *Major atelectasis/collapse*<br>• *Infiltrate (consolidation/alveolar opacity)*<br>• *Scarring/fibrosis/honeycombing*<br>• *Bronchial wall thickening*

		• *Bronchiectasis*<br>• *Emphysema/COPD/Bullae*<br>• *Edema*<br>• *Distortion of pulmonary architecture*
	Hilum<br>• *Left*<br>• *Right*	• *Enlarged non-calcified lymph nodes*<br>• *Enlarged calcified lymph nodes*<br>• *Hilar asymmetry*
	Pulmonary Arteries<br>• *Left*<br>• *Right*	• *Dilatation of pulmonary arteries*<br>• *Amputation of pulmonary arteries*
	Mediastinum<br>• *Left*<br>• *Right*	• *Enlargement*<br>• *Shift*<br>• *Calcification*<br>• *Hiatal hernia*<br>• *Asymmetry*<br>• *Surgical Clips*
	Aorta	• *Elongation*<br>• *Dilatation*<br>• *Calcification*<br>• *Kinking*<br>• *Prosthesis*
	Heart<br>• *Right atrium*<br>• *Left atrium*<br>• *Right ventricle*<br>• *Left ventricle*	• *Calcification*<br>• *Pericardial fat pad*<br>• *Dilatation*<br>• *Pacemaker*<br>• *Valve replacements*<br>• *Coronary stents*<br>• *Other cardiac assist devices*<br>• *Surgical clips*
	Upper Abdomen	• *Surgical clips*
	Bones<br>• *Fracture (old)*<br>• *Fracture (new)*	• *Jaw (if included)*<br>• *Scapulae*<br>• *Humeri*

	• *Lesions*<br>• *Degenerative changes*<br>• *Reduced bone mineral density*<br>• *Implants*<br>• *Scoliosis (spine)*	• *Claviculae*<br>• *Ribs*<br>• *Sternum*<br>• *Spine*

Supplemental Table S1: Evaluated diagnostic findings to predict all-cause mortality (training step 1a) and subclinical findings supplementing diagnostic findings to predict all-cause mortality (training step 1b)

Supplemental Table S2: Summary of baseline characteristics and risk factors of the subjects classified as high risk and low risk of dying by radiologists and the deep learning network irrespective of the true outcome.

	**Radiologists**			**DL CNN**			**Comparison Radiologists vs. DL CNN**	
	**high risk**	**low risk**		**high risk**	**low risk**			
**Variables**	*N (%) mean±SD*	*N (%)/mean±SD*	*p*	*N (%)/mean±SD*	*N (%)/mean±SD*	*p*	*p*	*p*
**Participants**	65	35		65	35		1.00	1.00
**Race**								
White	55 (84.6%)	31 (88.6%)	0.77	54 (83.1%)	32 (91.4%)	0.37	1.00	1.00
Black	8 (12.3%)	4 (11.4%)	1.00	10 (15.4%)	2 (5.7%)	0.21	0.80	0.67
Other	2 (3.1%)	0 (0%)	0.54	1 (1.5%)	1 (2.9%)	1.00	1.00	1.00
**Male Sex**	34 (52.3%)	21 (60.0%)	0.53	39 (60.0%)	16 (45.7)	0.21	0.48	0.38
**Age**	62.8±5.5	61.9±4.7	0.41	63.7±5.1	60.1±4.6	<0.001*	0.32	0.12
**Obesity (BMI≥30 kg/m2)**	17 (26.2%)	8 (22.9%)	0.81	19 (29.2%)	6 (17.1%)	0.23	0.85	0.77
**Smoking**								
Current	34 (52.3%)	21 (60.0%)	0.53	35 (53.9%)	20 (57.1%)	0.83	1.00	1.00
Former	31 (47.7%)	14 (40.0%)	0.53	30 (46.2%)	15 (42.9%)	0.83	1.00	1.00
**Diabetes**	10 (15.4%)	4 (11.8%)	0.77	13 (20.3%)	1 (2.9%)	0.02*	0.50	0.20
**Hypertension**	23 (35.4%)	11 (31.4%)	0.83	25 (38.5%)	9 (25.7%)	0.27	0.86	0.79
**History**								
Stroke	4 (6.2%)	2 (5.7%)	1.00	4 (6.2%)	2 (5.7%)	1.00	1.00	1.00
Myocardial infarction	12 (18.5%)	6 (17.7%)	1.00	13 (20.3%)	5 (14.3%)	0.59	0.83	0.75
Cancer	2 (3.1%)	3 (8.6%)	0.34	3 (4.6%)	2 (5.7%)	1.00	1.00	1.00
**CXR Findings**								
Pulmonary lesion	5 (7.7%)	0 (0%)	0.16	3 (4.6%)	2 (5.7%)	1.00	0.72	0.49
Emphysema	4 (6.2%)	0 (0%)	0.30	3 (4.6%)	1 (2.9%)	1.00	1.00	1.00
Cardiac enlargement	9 (13.9%)	0 (0%)	0.03*	8 (12.3%)	1 (2.9%)	0.16	1.00	1.00
Aortic pathology	3 (4.6%)	0 (0%)	0.55	3 (4.6%)	0 (0%)	0.55	1.00	1.00

Supplemental Table S2: Summary of baseline characteristics and risk factors of the subjects classified as high risk and low risk of dying by radiologists and the DL CNN. * indicates statistical significance; DL CNN=deep learning convolutional neural network.

Supplemental Table S3: Summary of baseline characteristics and risk factors of the subjects correctly classified (participants actually died/did not die) by radiologists and the deep learning network.

	Radiologists correctly classified risk			DL CNN correctly classified risk			Comparison Radiologists vs. DL CNN	
	high risk	low risk		high risk	low risk			
**Variables**	*N (%)/mean±SD*	*N (%)/mean±SD*	*p*	*N (%)/mean±SD*	*N (%)/mean±SD*	*p*	*p*	*p*
**Participants**	39	24		38	23		1.00	1.00
**Race**								
White	32 (82.1%)	23 (95.8%)	0.14	31 (81.6%)	23 (100%)	0.04*	1.00	1.00
Black	6 (15.4%)	1 (4.2%)	0.24	7 (18.4%)	0 (0%)	0.04*	0.77	1.00
Other	1 (2.6%)	0 (0%)	1.00	0 (0%)	0 (0%)	1.00	1.00	1.00
**Male Sex**	20 (51.3%)	13 (54.2%)	1.00	23 (60.5%)	11 (47.8%)	0.43	0.49	0.77
**Age**	63.9±5.2	61.9±4.7	0.14	64.4±4.9	60.1±4.7	0.001*	0.62	0.20
**Obesity (BMI≥30 kg/m2)**	10 (25.6%)	3 (12.5%)	0.34	12 (31.6%)	3 (13.0%)	0.13	0.62	1.00
**Smoking**								
Current	24 (61.5%)	13 (54.2%)	0.61	22 (57.9%)	10 (43.5%)	0.30	0.82	0.56
Former	15 (38.5%)	11 (45.8%)	0.61	16 (42.1%)	13 (56.5%)	0.30	0.82	0.56
**Diabetes**	6 (15.4%)	2 (8.3%)	0.70	8 (21.6%)	1 (4.4%)	0.13	0.56	1.00
**Hypertension**	16 (41.0%)	6 (25.0%)	0.28	16 (42.1%)	4 (17.4%)	0.06	1.00	0.72
**History**								
Stroke	3 (7.7%)	2 (8.3%)	1.00	3 (7.9%)	2 (8.7%)	1.00	1.00	1.00
Myocardial infarction	9 (23.1%)	3 (12.5%)	0.35	11 (29.7%)	4 (17.4%)	0.37	0.61	0.70
Cancer	2 (5.1%)	2 (8.3%)	0.63	3 (7.9%)	2 (8.7%)	1.00	0.68	1.00
**CXR Findings**								
Pulmonary lesion	4 (10.3%)	0 (0%)	0.29	2 (5.3%)	0 (0%)	0.52	0.68	1.00
Emphysema	4 (10.3%)	0 (0%)	0.29	3 (7.9%)	0 (0%)	0.28	1.00	1.00
Cardiac enlargement	6 (15.4%)	0 (0%)	0.07	6 (15.8%)	1 (4.4%)	0.24	1.00	1.00
Aortic pathology	2 (5.1%)	0 (0%)	0.52	2 (5.3%)	0 (0%)	0.52	1.00	1.00

Supplemental Table S3: Summary of baseline characteristics and risk factors of the subjects correctly classified by radiologists and the DL CNN * indicates statistical significance; DL CNN=deep learning convolutional neural network.

Supplemental Figures

Frequency	Event rate	Rating	HR (95% CI)	p value
7%	28.6% (2/7)	Very low	Reference	NA
16%	37.5% (6/16)	Low	1.41 (0.28-7.01)	0.67
40%	37.5% (15/40)	Moderate	1.27 (0.29-5.58)	0.75
30%	66.6% (20/30)	High	3.01 (0.70-12.93)	0.14
7%	100% (7/7)	Very high	5.96 (1.23-28.78)	0.02

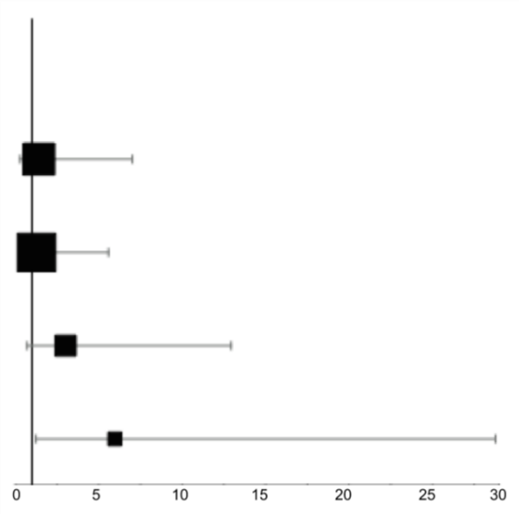

Supplemental Figure S1: Ratings for 6-year mortality by radiologists in the tuning dataset. HR=hazard ratio; CI=confidence interval.
